# Synthesis and Structure of the Inclusion Complex {NdQ[5]K@Q[10](H_2_O)_4_}·4NO_3_·20H_2_O

**DOI:** 10.3390/molecules22071147

**Published:** 2017-07-09

**Authors:** Li Xia Chen, Jing Lan Kan, Hang Cong, Timothy J. Prior, Zhu Tao, Xin Xiao, Carl Redshaw

**Affiliations:** 1 Key Laboratory of Macrocyclic and Supramolecular Chemistry of Guizhou Province, Guizhou University, Guiyang 550025, China; chenlixia19@163.com (L.X.C.); ecnuc@163.com (H.C.); gzutao@263.net (Z.T.); 2 College of Chemistry, Chemical Engineering and Materials Science, Collaborative Innovation Center of Functionalized Probes for Chemical Imaging in Universities of Shandong, Key Laboratory of Molecular and Nano Probes, Ministry of Education, Shandong Normal University, Jinan 250014, China; juha.kiviluoma@vtt.fi; 3 Department of Chemistry, School of Mathematics and Physical Sciences, University of Hull, Hull HU6 7RX, UK; t.prior@hull.ac.uk

**Keywords:** inclusion complex, cucurbit[10]uril, cucurbit[5]uril, neodymium, potassium, structure

## Abstract

Heating a mixture of Nd(NO_3_)_3_·6H_2_O, KCl, Q[10] and Q[5] in HCl for 10 min affords the inclusion complex {NdQ[5]K@Q[10](H_2_O)_4_}·4NO_3_·20H_2_O. The structure of the inclusion complex has been investigated by single crystal X-ray diffraction and by X-ray Photoelectron spectroscopy (XPS).

## 1. Introduction

Cucurbit[*n*]urils have attracted increased attention over the last decade or so, with much of the interest resulting from their recognition properties [1]. Their varied chemistry and structures together with potential applications have been discussed in a number of recent reviews. We and others have been interested in their host–guest chemistry, as well as the application of their recognition properties [2,3,4,5,6]. We have also been attracted by a number of reports in which a smaller member of the Q[*n*] family can be trapped in the cavity of a larger member of the series. Such inclusion complexes for Q[5] and Q[10] were first reported by Day and coworkers [7]. In particular, the gyroscane (gyroscope-like) species [Cl@Q[5]·(OH_2_)_2_]@Q[10] was structurally characterized and could be isolated on 65 g scale. Later studies showed it was possible to isolate free Q[10] from this inclusion complex [8]. More recently, Liu et al. employed a template route to access the inclusion complex [H_2_O@Q[5]K_2_@Q[10]]Cl_2_(HCl)(H_2_O)_37_ [9]. We were intrigued by the stabilizing ability of the potassium ions in this structure and have embarked upon a programme to investigate other cucurbit[*n*]uril inclusion complexes, particularly species in which smaller Q[*n*] reside within much larger Q[*n*]s.

## 2. Results and Discussion

**Figure 1:**
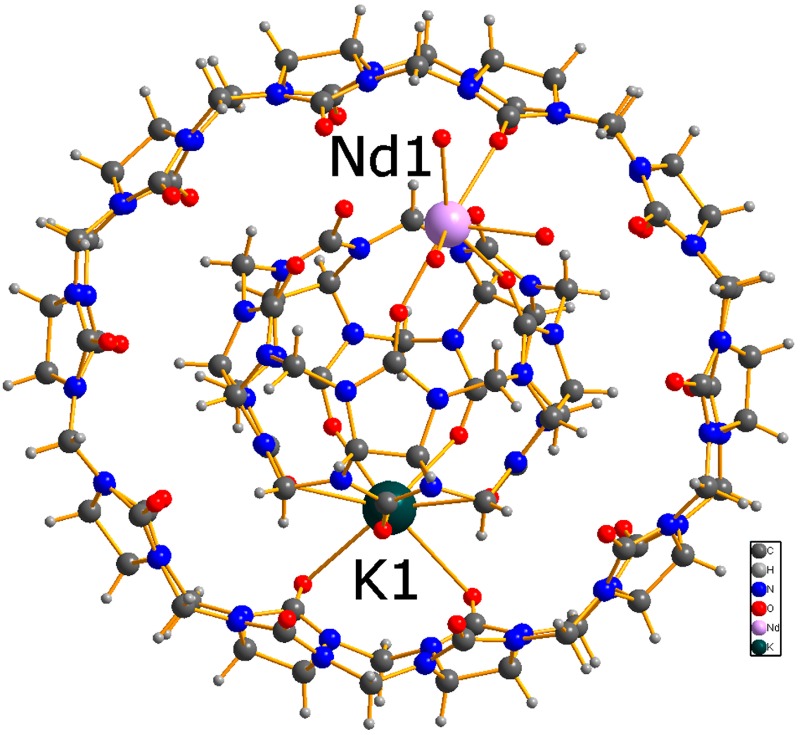
Molecular structure of the inclusion complex {NdQ[5]K@Q[10](H_2_O)_4_}·4NO_3_·20H_2_O as viewed from the top.

A mixture containing Q[6], Q[8] and Q[5]@Q[10] was generated by heating glycoluril, paraformaldehyde and hydrochloric acid at 100 °C for 6 h [10,11]. Subsequent treatment with HCl and amantadine resulted in isolation of amantadines-Q[10], from which pure Q[10] can be obtained on work-up using DMSO [12]. An equimolar mixture of Q[10] and Q[5] was dissolved in HCl (1M), to which neodynium nitrate and potassium chloride were added. Following brief (10 min) heating (80 °C), the solution was left to stand at ambient temperature under air. On prolonged standing (5 weeks), single crystals suitable for X-ray diffraction were obtained in about 12% yield. The molecular structure is shown in Figure 1, and it can be seen that as for the structure reported by Liu et al. [9], the Q[5] is contained within the Q[10]. Here however, as well as being locked in place by a K^+^ ion, the rigidity of the structure (versus gyroscane) is enhanced by coordination of Nd(H_2_O)_4_^3+^. Furthermore, between the Q[*n*]s there are four nitrate ions and approximately 20 additional unbound water molecules present (some of which are identified by the PLATON Squeeze routine). The Q[5] is not co-planar with the Q[10]. Although their centroids are very close (~0.167 Å apart), the Q[5] is rotated so that the plane of this molecule is inclined at 62° to the plane of the Q[10].

**Figure 2:**
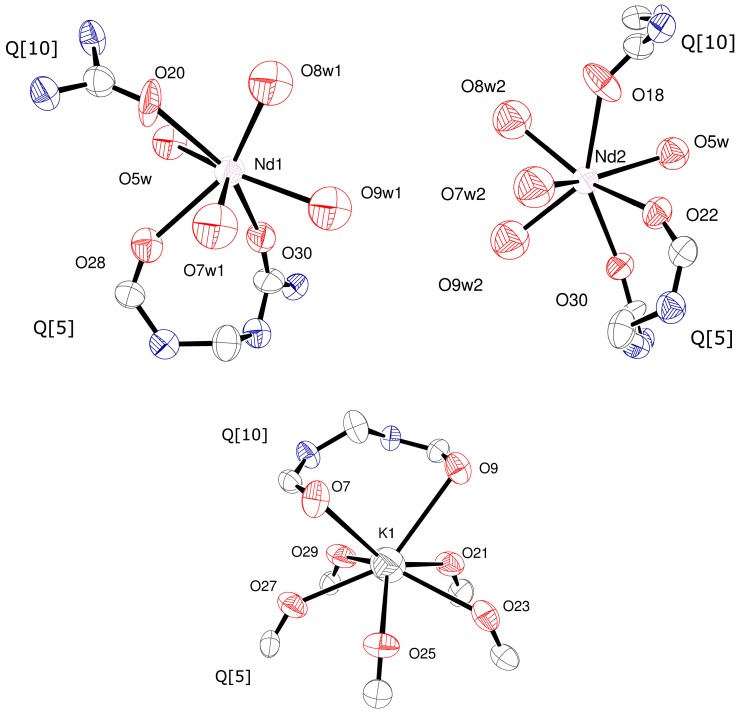
Coordination environments around Nd(1), Nd(2) and K(1).

The view of the inclusion complex given in Figure S1 (see ESI) emphasizes how the neodymium centre sits above the central mean plane (‘belt’) of the Q[10] by approximately 1.63(2) Å. This neodymium centre is disordered over two positions Nd1 (56.33(18)% occupied) and Nd2 (43.67(18)% occupied). By contrast, the potassium ion is embedded in the structure. The coordination geometries about each Nd centre and K(1) are illustrated in Figure 2. The five shorter K–O bonds (<2.9 Å) are to the Q[5], whilst the other two longer K–O bonds (>3.1 Å) are to the Q[10]; for the Liu structure, the K–O bonds to the Q[5] are <ca. 2.9 Å and the longer bonds to Q[10] >2.9 Å [9]. For each of the two 7-coordinate Nd positions, the bond lengths to the Q[*n*]s and those between Nd and H_2_O are similar at ca. 2.4 to 2.5 Å. Dimensions across the Q[10] (for carbonyls across the length of the Q[10]) are in the range 11.39(2)–13.82(2) Å (see Figure S2, ESI); for the Liu structure, the range is 11.9–13.7 Å [9].

We note that in the literature a number of Nd complexes have been previously reported for Q[*n*]s, where n = 5 to 8 [13,14,15,16,17,18,19,20,21], some of which are heterometallic capped species involving K^+^ ions [22,23,24]. Of particular note here are the Q[5] heterobimetallic complexes {[NdKQ[5]Cl]Cl}(H_2_O)_3_}Cl_2_·12.5H_2_O [22] and {[Nd(H_2_O)_3_][(NO_3_)@Q[5]][K(H_2_O)][Nd(H_2_O)_3_(NO_3_)_4_]} ·2(NO_3_)·8H_2_O [23]. In the first of these heterobimetallic complexes, where the Q[5] encapsulates a chloride anion, the Nd centre is nine-coordinate (5 carbonyls at an average distance of 2.531 Å, 3 waters at an average distance of 2.516 Å and the internal chloride, whilst the K^+^ is bound by an internal and external chloride as well as 5 oxygen centres of the Q[5]. By contrast, in the second complex, the K^+^ coordinates to ten oxygen atoms (5 carbonyls at an average distance of 2.783 Å, H_2_O at 3.257 Å and 4 from two nitrates). Furthermore, in this second structure, the Nd centre coordinates to nine oxygen atoms (5 carbonyls with distances in the range 2.511–2.570 Å, 3H_2_O at an average distance of 2.448 Å and a nitrate). The structures of the two inclusion complexes (herein and the Liu complex) indicate that if the ‘external’ atoms are part of a larger Q[*n*] system, then their coordination to that either K or Nd centres of a Q[5] results in this Q[*n*] system being held in a fixed position relative to the inner Q[5].

**Figure 3:**
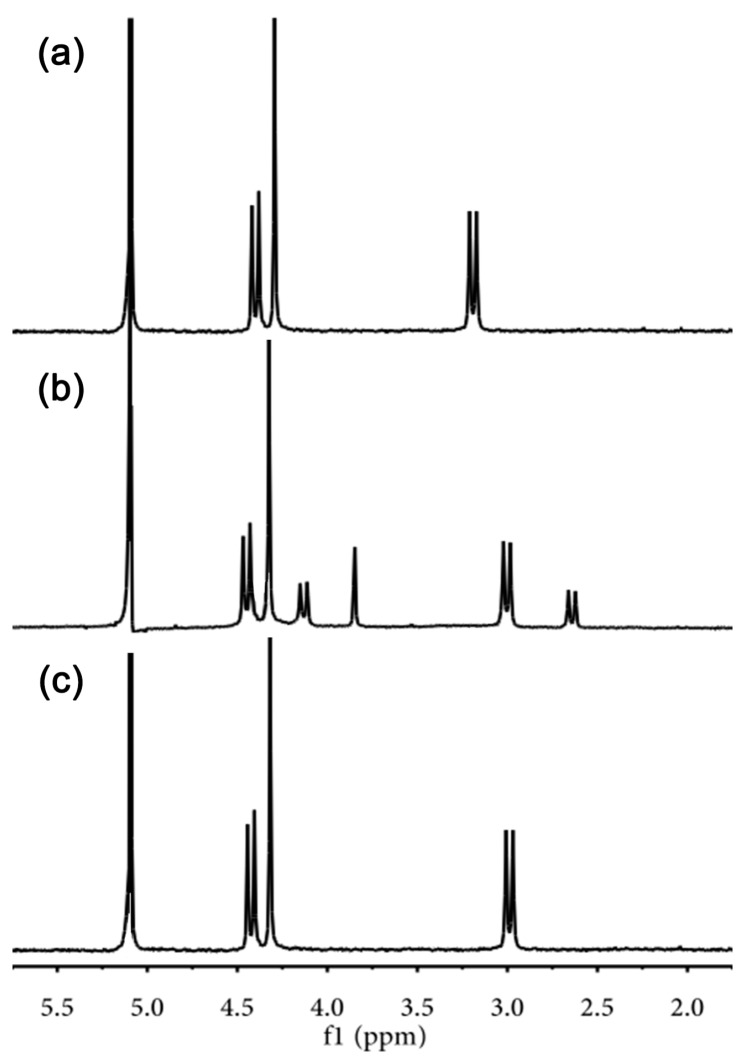
^1^H NMR spectra in 4 M DCl: (**a**) Q[5]; (**b**) Q[5]@Q[10]; (**c**) Q[10].

The structure of the inclusion complex is retained in solution as evidenced by the ^1^H NMR spectrum (see Figure 3), for which the methylene peaks all appear as doublets (i.e., non-equivalent). Whilst the positions of the peaks associated with Q[10] remain relatively unchanged, those of Q[5] tend to be shifted somewhat upfield.

**Figure 4:**
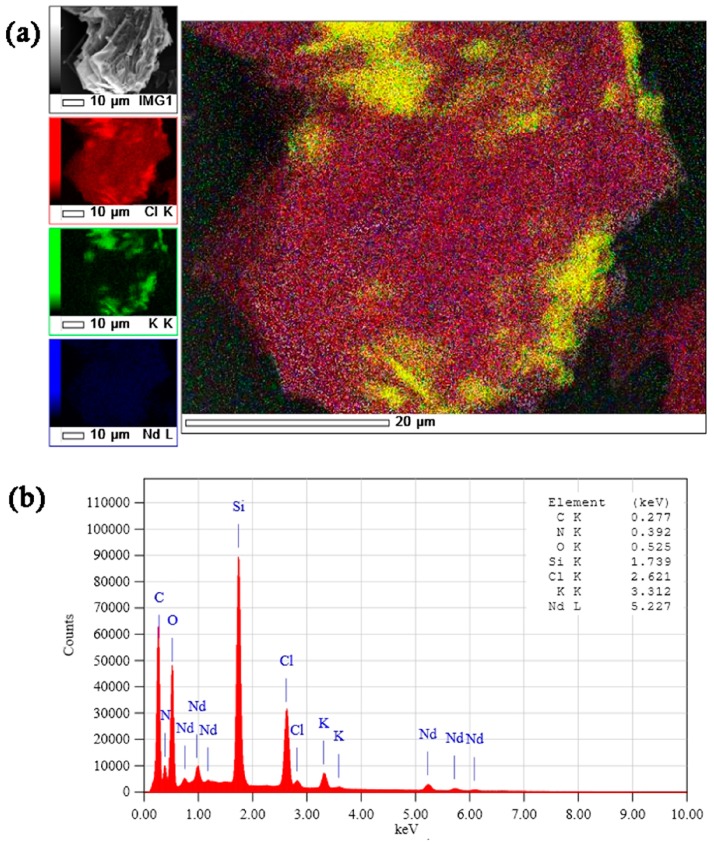
The XPS spectra of the inclusion complex. (**a**) the X-ray photoelectron spectra of {NdQ[5]K@Q[10](H2O)4}·4NO_3_·20H_2_O and through the elemental mappings; (**b**) in terms of the cations, the obtained crystals contained 50% K and 50% Nd, suggesting the X-ray photoelectron spectra are consistent with the single crystal structure.

The X-ray photoelectron spectra of {NdQ[5]K@Q[10](H_2_O)_4_}·4NO_3_·20H_2_O are shown in Figure 4, and through the elemental mappings (Figure 4a), it is apparent that there is a homogeneous distribution of K and Nd in the sample. In addition, in terms of the cations, the obtained crystals contained 50% K and 50% Nd, suggesting the X-ray photoelectron spectra are consistent with the single crystal structure (Figure 4b), i.e., 1:1 K:Nd in the crystal.

## 3. Materials and Methods

### 3.1. General

All the chemicals were commercially available and used as received without further purification. Glycoluril was synthesized by our laboratory. 

### 3.2. Measurements

XPS images were obtained using a JEOL JSM-6700F field-emission scanning electron microscopy (JOEL Ltd, Tokyo, Japan). Elemental analysis was performed on an Elementar Vavio El III (Elementar Analysensysteme GmbH, Frankfurt, Germany).

### 3.3. Crystal Structure Determination

A suitable single crystal (~0.2 × 0.2 × 0.1 mm^3^) was taken up in paraffin oil and mounted on a Bruker SMART Apex II CCD diffractometer (Bruker AXS, Madison, WI, USA) equipped with a graphite monochromator Mo-Kα (λ = 0.71073 Å, μ = 0.492 mm^−1^) radiation source operating in the ω-scan mode. Data were corrected for Lorentz and polarization effects using SAINT (Bruker AXS, Madison, WI, USA). The structure was solved by direct methods, and were refined against F^2^ using the full-matrix least-squares method using SHELXL-2016. [25] All non-hydrogen atoms were refined anisotropically. Carbon-bound hydrogen atoms were introduced at calculated positions, and were treated as riding atoms with an isotropic displacement parameter equal to 1.2 times that of the parent atom. The complexes present are very large and contain over 200 unique non-hydrogen atoms and there are portions of disordered guest molecules. The X-ray scattering drops away very rapidly beyond about 1 Å resolution and data beyond this limit were not employed in the refinement as these are essentially noise. The PLATON Squeeze routine was employed and showed that the structure contained four pockets within the unit cell that contained disordered guest. The total volume of these pockets was ~2280 Å^3^ and this corresponded to an electron count of 4 × 126e. For charge balancing, an additional disordered nitrate ion is required and the remaining space is occupied by three water molecules [26].

Details of the crystal parameters, data collection conditions, and refinement parameters for the compound are summarized in Table 1. In addition, the crystallographic data for the reported structures were deposited at the Cambridge Crystallographic Data Centre with the following supplementary publication numbers: CCDC-1554311.

### 3.4. Synthesis of Complex 1

A mixture of glycoluril (50 g, 0.35 mol), paraformaldehyde (26.2 g, 0.86 mol), and concentrated HCl (100 mL) was stirred at room temperature for 1 h and then heated at 100 °C for 6 h [7,8]. The resulting solution was cooled to room temperature and precipitated by pouring into MeOH (500 mL) to yield a crude pale yellow solid. The precipitate was extracted three times with 50 mL of water. The filtered residue (45 g) containing Q[6], Q[8] and Q[5]@Q[10] was dissolved in 6 M HCl (300 mL) and amantadines (7.5 g) were added to induce precipitation (1.1 g, amantadines-Q[10]). The precipitate was filtered and dried, and refluxed with DMSO (50 mL) for 30 min. The precipitate was filtered then refluxed with MeOH (50 mL) for 30 min, filtered and dried to yield ~1.0 g of pure Q[10] [9]. Q[10] (1.66 g, 0.001 mol) and Q[5] (0.83 g, 0.001 mol) was then dissolved in 1 M hydrochloric acid (100 mL). To this solution was added Nd(NO_3_)_3_·6H_2_O (4.38 g, 0.01 mol) and potassium chloride (0.75 g, 0.01 mmol). The mixture was heated at 80 °C for 10 min, then left to stand at room temperature in an open beaker. After five weeks, X-ray quality colorless crystals of the title complex were obtained in a yield of 12.1%. Anal. calcd. For C_90_H_138_N_64_O_66_KNd (%): C, 33.59; H, 0.03; N, 27.86, Found: C, 33.41; H, 0.09; N, 27.49.

## 4. Conclusions

The inclusion complex {NdQ[5]K@Q[10](H_2_O)_4_}·4NO_3_·20H_2_O has been isolated from a mixture comprising of Nd(NO_3_)_3_·6H_2_O, KCl, Q[10] and Q[5] in HCl. A crystal structure determination reveals that the Q[5] is held firmly within a Q[10] by coordination of potassium and neodymium ions; the structure is maintained in solution.

## Figures and Tables

**Table 1:** Crystal data and structure-refinement details for complex **1**.

Compound	1
Empirical formula	C90 H90 K N64 Nd O66
Formula weight	3313.58
Crystal system	Orthorhombic
Space group	*P c a 2_1_*
*a*, Å	18.998(7)
*b*, Å	29.043(10)
*c*, Å	26.562(9)
*α*, deg	90
*β*, deg	90
*γ*, deg	90
*V*, Å^3^	14656(9)
Z	4
Dcalcd, g cm^−3^	1.449
T, K	293(2)
*μ*, mm^−1^	0.492
Unique reflns	14709
Obsd reflns	10294
Params	1774
Rint	0.1265
R[I > 2σ(I)] ^a^	0.0829
wR[I > 2σ(I)] ^b^	0.2132
R(all data)	0.1178
wR(all data)	0.2466
GOF on F2	1.020

^a^ R_1_ = Σ||F_o_| − |F_c_||/Σ|F_o_|; ^b^ wR_2_ = |Σw(|F_o_|^2^ − |F_c_|^2^)|/Σ|w(F_o_)^2^|^1/2^, where w = 1/[σ^2^(F_o_^2^) + (aP)^2^ + bP]; P = (F_o_^2^ + 2F_c_^2^)/3.

## Supplementary Materials

The following are available online. Figure S1: Structure of the inclusion complex {NdQ[5]K@Q[10](H_2_O)_4_}·4NO_3_·20H_2_O as viewed from the side ; Figure S2: Dimensions of the Q[10]. The distances are in Å; the inner Q[5] and other atoms have been removed for clarity.
