# Erratum: Chen, L.X., et al. Synthesis and Structure of the Inclusion Complex {NdQ[5]K@Q[10](H_2_O)_4_}•4NO_3_•20H_2_O. *Molecules* 2017, *22*, 1147

**DOI:** 10.3390/molecules24020328

**Published:** 2019-01-17

**Authors:** Li Xia Chen, Jing Lan Kan, Hang Cong, Timothy J. Prior, Zhu Tao, Xin Xiao, Carl Redshaw

**Affiliations:** 1 Key Laboratory of Macrocyclic and Supramolecular Chemistry of Guizhou Province, Guizhou University, Guiyang 550025, China; chenlixia19@163.com (L.X.C.); ecnuc@163.com (H.C.); gzutao@263.net (Z.T.); 2 College of Chemistry, Chemical Engineering and Materials Science, Collaborative Innovation Center of Functionalized Probes for Chemical Imaging in Universities of Shandong, Key Laboratory of Molecular and Nano Probes, Ministry of Education, Shandong Normal University, Jinan 250014, China; kanjinglan@163.com; 3 Department of Chemistry, School of Mathematics and Physical Sciences, University of Hull, Hull HU6 7RX, UK; t.prior@hull.ac.uk

The authors wish to make the following correction to their paper [1]:

We found that in the Authorship section, below the second address, a mistake exists on the published page. The e-mail, which is stated as “juha.kiviluoma@vtt.fi”, should be corrected to “kanjinglan@163.com”. The changes do not affect the scientific results. The manuscript will be updated and the original will remain online on the article webpage, with a reference to this correction.
