# Supplementary material for: Synthesis and Structure of the Inclusion Complex {NdQ[5]K@Q[10](H2O)4}·4NO3·20H2O
Source: Molecules. 2017 Jul 9;22(7):1147. doi: 10.3390/molecules22071147 (PMC6152283; doi:10.3390/molecules22071147)
Supplement: Supplementary file 1 [file molecules-22-01147-s001.pdf]

Electronic Supplementary Information

Synthesis and structure of the inclusion complex $\{NdQ[5]K@Q[10](H_2O)_4\}\cdot 4NO_3\cdot 20H_2O$

Li Xia Chen,[a] Jing Lan Kan,[b] Hang Cong,[a] Timothy J. Prior,[c] Zhu Tao,[a] Xin Xiao,*[a] and Carl Redshaw[c*]

[a] Key Laboratory of Macrocyclic and Supramolecular Chemistry of Guizhou Province, Guizhou University, Guiyang 550025, China

[b] College of Chemistry, Chemical Engineering and Materials Science, Collaborative Innovation Center of Functionalized Probes for Chemical Imaging in Universities of Shandong, Key Laboratory of Molecular and Nano Probes, Ministry of Education, Shandong Normal University, Jinan 250014, China.

[c] School of Mathematics and Physical Sciences, University of Hull, Hull HU6 7RX, U.K.

Contents

Figure S1. Structure of the inclusion complex $\{NdQ[5]K@Q[10](H_2O)_4\}\cdot 4NO_3\cdot 20H_2O$ as viewed from the side.

Figure S2. Dimensions of the Q[10]. The distances are in Å; the inner Q[5] and other atoms have been removed for clarity.

Figure S1. Structure of the inclusion complex $\{NdQ[5]K@Q[10](H_2O)_4\}.4NO_3.20H_2O$ as viewed from the side.

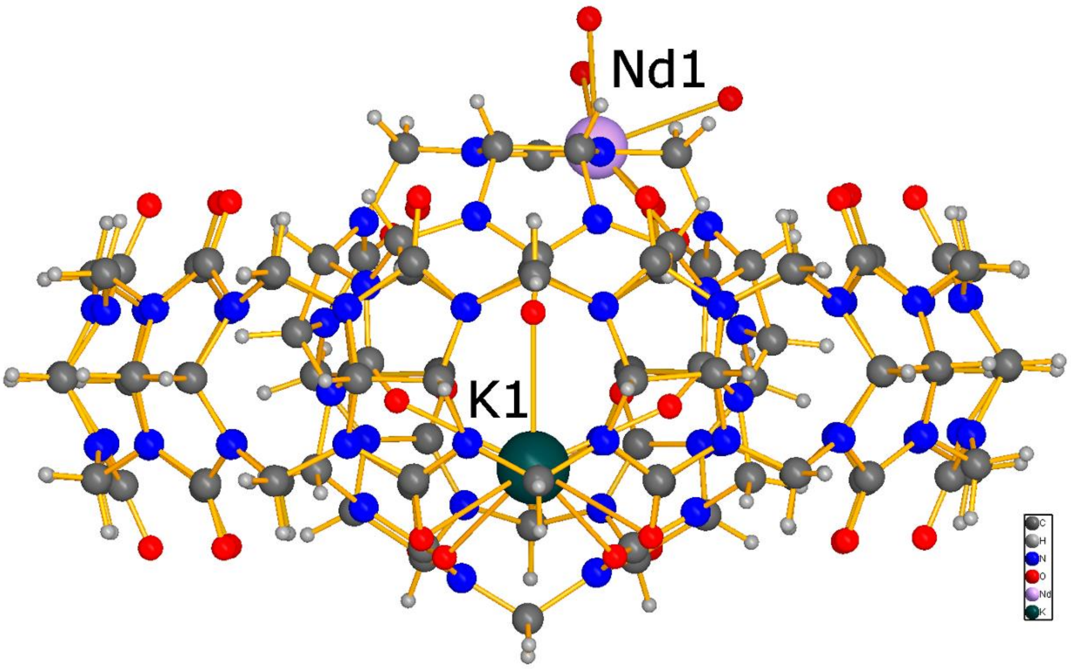

Figure S2. Dimensions of the Q[10]. The distances are in Å; the inner Q[5] and other atoms have been removed for clarity.

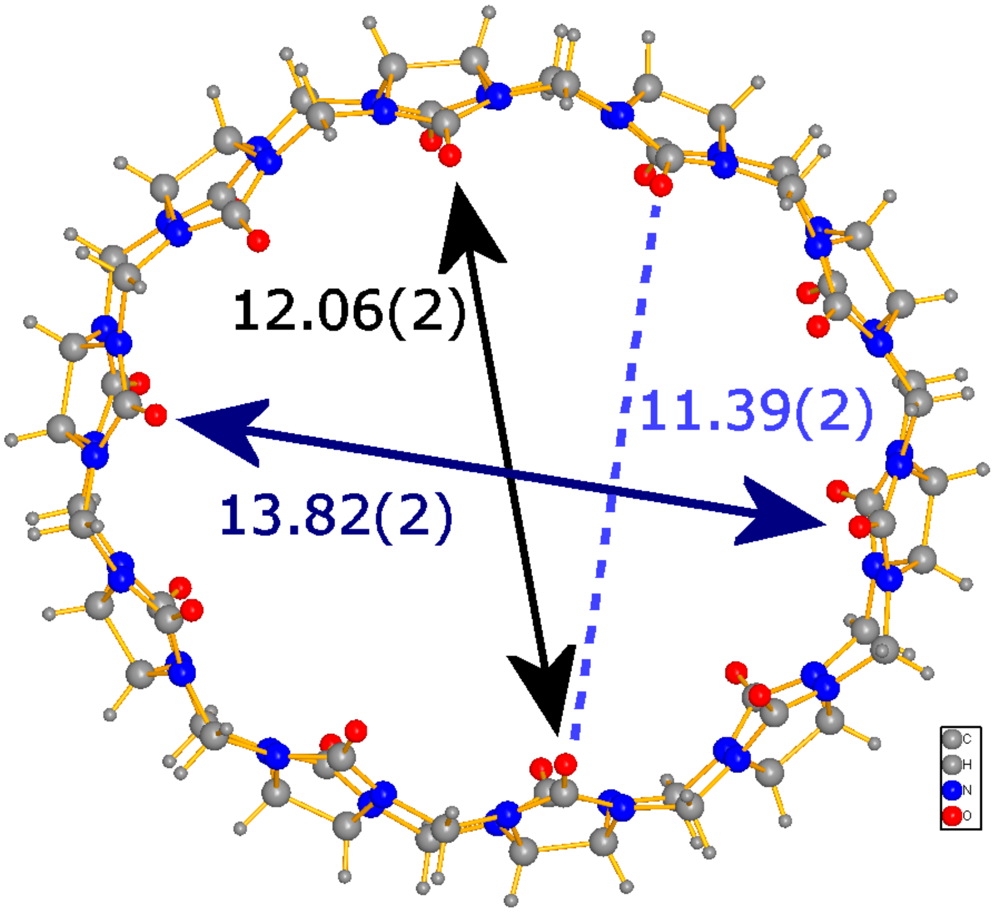
12.06(2)
11.39(2)
13.82(2)
C
H
N
O
